# Risk of Osteonecrosis of the Jaw in Patients Treated with Zoledronic or Alendronic Acid: A Systematic Review

**DOI:** 10.3390/medicina61071159

**Published:** 2025-06-26

**Authors:** Aine Jakonyte, Egle Gustainyte, Zygimantas Petronis, Aviad Hafizov, Audra Janovskiene, Dainius Razukevicius

**Affiliations:** 1 Department of Odontology, Lithuanian University of Health Sciences (LSMU), LT-50161 Kaunas, Lithuania; egle.gustainyte@stud.lsmu.lt (E.G.); aviad.hafizov@stud.lsmu.lt (A.H.); 2 Department of Maxillofacial Surgery, Faculty of Odontology, Medical Academy, Lithuanian University of Health Sciences (LSMU), LT-50161 Kaunas, Lithuania; zygimantas.petronis@lsmu.lt (Z.P.); audrsuli0210@kmu.lt (A.J.); dainius.razukevicius@lsmu.lt (D.R.)

**Keywords:** alendronate, bisphosphonate-associated osteonecrosis of the jaw, osteoporosis, zoledronic acid

## Abstract

*Background and Objectives:* Bisphosphonates (BP) like zoledronic acid (ZA) and alendronic acid (AA) are used for osteoporosis (OP) or other bone-related conditions as well as to prevent the spread of metastases and in rheumatoid arthritis treatment. However, they have been associated with an increased risk of osteonecrosis of the jaw (ONJ). This systematic review aimed to assess the incidence and risk of ONJ in osteoporotic patients treated with ZA or AA and evaluate the impact of treatment duration. *Material and Methods:* The systematic literature review was conducted following PRISMA guidelines. The keywords “Zoledronic acid,” “Alendronic acid,” “Osteoporosis,” and “Osteonecrosis” were searched in PubMed and ScienceDirect databases. Selection criteria included studies on humans written in English, published from 2014. The systematic review protocol was registered in the PROSPERO register under the following number: CRD42024587046. *Results:* A total of 7 studies with 98,717 osteoporotic patients met the criteria, showing a higher ONJ incidence with ZA than AA. Six studies linked longer BP use to increased ONJ risk, which quadrupled after 5 years of AA use. A positive correlation was found between BP use (≥3 years) and ONJ in OP patients, primarily affecting females over 60. ONJ appeared after 1 year with AA, increasing over time, while ZA-related ONJ emerged as early as 5 months with a higher overall incidence. *Conclusions:* ZA poses a higher ONJ risk and incidence and earlier onset than AA, occurring within 5 months versus 1 year for AA. These findings emphasize the need for careful monitoring, especially in long-term BP therapy with additional risk factors.

## 1. Introduction

Osteoporosis (OP) is a chronic bone disease characterized by reduced bone mineral density and microarchitectural deterioration, leading to increased fragility and fracture risk [1]. While OP affects various age groups, it is most prevalent in postmenopausal women and elderly individuals [2]. According to the International OP Foundation, approximately 1 in 3 women and 1 in 5 men over the age of 50 will experience osteoporotic fractures in their lifetime [3]. OP is often asymptomatic until a fracture occurs, commonly affecting the hip, spine, and wrist, with significant morbidity and mortality risks [4]. Given its high prevalence, long-term consequences, and increasing burden on healthcare systems worldwide, osteoporosis remains a major public health concern.

Although OP primarily impacts long bones, it can also affect the jawbone, leading to dental complications such as tooth loss, impaired healing, and challenges with dental implants [5]. A major concern in OP treatment is osteonecrosis of the jaw (ONJ), a severe condition associated with bisphosphonate (BP) therapy, which is widely used to manage OP as well as to prevent the spread of metastases and in rheumatoid arthritis treatment [6]. Several studies have investigated the relationship between BP therapy and the development of ONJ, particularly focusing on drug potency, administration route, and patient risk factors.

Zoledronic acid (ZA) and alendronic acid (AA) are among the most commonly prescribed BP, though they differ in their potency, route of administration, and clinical use. ZA is significantly more potent and typically administered as an annual intravenous infusion, improving adherence, especially in oncology patients for managing bone metastases and hypercalcemia of malignancy. However, ZA carries risks such as infusion-related adverse effects, including ONJ [7]. In contrast, AA is usually taken orally on a weekly basis and is commonly prescribed for the treatment of osteoporosis and other metabolic bone diseases such as Paget’s disease, bone metastases, and multiple myeloma. It offers a more accessible treatment option but requires strict adherence to dosing instructions in order to minimize gastrointestinal side effects and ONJ risk [8]. The risk of osteonecrosis is increased in conjunction with oral surgery due to incorrect prosthetic planning or trauma to the oral cavity as well.

Although both drugs effectively reduce bone resorption, they act by inhibiting the enzyme farnesyl pyrophosphate synthase in the mevalonate pathway, which impairs osteoclast function and survival [7]. ZA has a higher potency and prolonged skeletal retention; this leads to its increased therapeutic impact but is also linked to a higher risk of medication-related osteonecrosis of the jaw (MRONJ), particularly in cancer patients receiving long-term treatment [9]. It is important to mention that it is necessary to indicate that the patient’s medical history must be accurate to promptly identify patients who may be at risk of osteonecrosis, even if they do not suffer from osteoporosis. ZA and AA’s differences in administration route, dosing frequency, and pharmacokinetics are key factors influencing both patient compliance and potential adverse effects, especially the risk of ONJ. However, the specific role of treatment duration in influencing ONJ risk remains insufficiently understood, especially when comparing ZA and AA use in osteoporotic patients. Therefore, this systematic review aims to address this gap by evaluating how the duration of BP use, specifically ZA and AA, influences the incidence of ONJ in osteoporotic patients.

This systematic review aims to evaluate how the duration of BP use, specifically ZA and AA, influences the incidence of ONJ in osteoporotic patients. The analysis focuses on identifying risks associated with treatment duration and assessing which BP poses a higher likelihood of ONJ development. By synthesizing current findings, this review seeks to provide clinically relevant insights into the safe use of these medications and the potential risks associated with long-term treatment. The main question analyzed in this systematic review is as follows: how does the length of BP therapy (ZA or AA) affect the risk of developing ONJ in patients with osteoporosis?

Through a comprehensive synthesis of current evidence, this study seeks to offer clinically relevant insights, inform dental and medical practice, and improve patient safety in long-term BP therapy.

## 2. Materials and Methods

### 2.1. Protocol and Registration

This systematic review was conducted following the PRISMA (Preferred Reporting Item for Systematic Review and Meta-Analyses) guidelines [10]. The methods of the analysis and inclusion criteria were specified in advance and documented in a protocol, accessible through the following link: https://www.crd.york.ac.uk/prospero/ (accessed on 27 December 2023).

The systematic review protocol was registered in the PROSPERO: CRD42024587046.

### 2.2. Focus Question

Based on the PICO model (Table 1), the following question was developed: how does the incidence, risk, and treatment duration of ZA or AA affect the development of ONJ in osteoporotic patients?

### 2.3. Information Sources

The search for articles was conducted from 24 November 2023 to 6 January 2025 (the last date of the database search was 6 January 2024) in the following databases: PubMed and ScienceDirect.

### 2.4. Search

Following the PRISMA guidelines, an advanced search was conducted across multiple resource databases. Various keywords and their combinations were applied using Boolean operators, such as AND and OR, to refine online research. The primary search terms included the following: “zoledronate” OR “zoledronic acid” OR “alendronate” OR “alendronic acid” AND “osteonecrosis” OR “ONJ” AND “jaw”.

### 2.5. Selection of Studies

The titles of the resulting articles were reviewed independently by two reviewers (A.J. and E.G.) based on the exclusion and inclusion criteria. A third reviewer (Z.P.) ensured that there were no typographical errors. Articles that met the selection criteria underwent a full-text analysis. The reviewers independently assessed the results, and any disagreements were resolved through discussion with the senior investigator (Z.P.). To ensure consistency among reviewers, Cohen’s kappa coefficient (κ) was calculated on a random sample of 10% of publications to verify inter-rater reliability for the abstract and title screenings.

### 2.6. Types of Publications

This systematic review included retrospective cohort studies, prospective studies and observational studies since 2014 investigating the incidence, risk and the impact of treatment duration of ZA or AA on ONJ development in OP patients. In this systematic review retrospective, prospective, and observational studies were compared to ensure a comprehensive review of the available literature. Due to the limited number of studies on this topic, restricting the study design would have significantly narrowed the scope. Authors acknowledge that retrospective studies carry a higher risk of bias regarding temporality, and this factor was considered during the quality assessment process.

### 2.7. Inclusion and Exclusion Criteria

To ensure relevance, quality, and reliability of the study, specific inclusion and exclusion criteria were applied during the selection process. These criteria helped to identify appropriate participants, studies and data, that meet the research objectives while excluding those that could introduce bias or confounding factors. 

Inclusion Criteria

- Type of publication: retrospective cohort studies, prospective studies. and observational studies
- Studies examining the incidence, risk and the impact of treatment duration of ZA or AA on the development of ONJ.
- Studies were required to confirm both OP diagnosis and ONJ occurrence, ensuring that the findings were relevant to the target patient population.
- Patients diagnosed with a rheumatologic condition or cancer who are prescribed ZA.
- Research conducted with human subjects.
- Studies published in English within the last 10 years.
- Access to the full text of the article.

Exclusion Criteria

- Type of publications: meta-analyses, systematic reviews, conference abstracts, case reports, or case series, as these do not provide original patient data.
- Research conducted on animals or in vitro/ex vivo models.
- Studies involving patients diagnosed with Paget’s disease or malignant neoplasm.
- Studies involving patients receiving denosumab or other bisphosphonates (except ZA or AA).
- Studies involving minors.
- Studies that lacked sufficient data or whose authors could not be contacted for clarification.

### 2.8. Sequential Search Strategy

The study selection process was conducted in two stages using the Rayyan^®^ (Qatar Computing Research Institute) platform (accessed on 29 December 2023). Firstly, titles and abstracts of the identified studies were screened. Articles that failed to meet the selection criteria or were found to be duplicates were removed from consideration. Secondly, full-text versions of the selected studies were reviewed in detail to determine their eligibility based on the predefined inclusion and exclusion criteria. All exclusions were made strictly in accordance with the predefined inclusion/exclusion criteria to ensure transparency and reproducibility of the selection process.

### 2.9. Data Extraction and Items

The data were extracted independently from the studies and categorized into variables based on the aims and themes of the present review.

The following data was extracted from each included study:

- Author;
- Type of publication;
- Sample size;
- Type of BP;
- Number of subjects;
- Method of assessment;
- Results;
- Conclusions;
- Follow-up.

### 2.10. Risk of Bias Within Studies

All included studies were assessed for the quality of included retrospective cohort studies to detect risk of bias. The quality of the studies was evaluated by two independent authors. The tool used for assessing the risk of systematic errors in research was selected according to the recommendations published by Ma et al. [11]. The risk of bias in each included publication was evaluated using The Joanna Briggs Institute (JBI) Critical Appraisal Checklist for cohort studies.

The JBI Critical Appraisal Checklist for cohort studies (Table 2) was applied to assess the methodological quality of the studies that satisfied the inclusion criteria. Each criteria was evaluated by selection of “Yes”, “No”, “Unclear” or “Not applicable”.

### 2.11. Statistical Analysis

Article citations were managed using Zotero^®^ version 6.0.37 (George Mason University, USA) reference manager software. The agreement level between the two raters in selecting abstracts and studies to full-text analysis was assessed using Cohen’s kappa coefficient (κ).

## 3. Results

### 3.1. Study Selection

A search of the PubMed and ScienceDirect databases, using a combination of keywords, yielded 526 articles. After duplicates were excluded, 374 articles remained. During the initial stage of selection, the articles titles and abstracts were reviewed; therefore, inclusion criteria were applied, and 11 articles remained. Four [12,13,14,15] studies were excluded. Although these studies were not included in the final quantitative synthesis due to methodological limitations, their findings were taken into account during qualitative assessment to ensure a balanced review.

During the full-text screening process, four studies were excluded due to not meeting the predefined inclusion criteria. The study by Kim et al. [12] was excluded because it examined alendronate and raloxifene, while the inclusion criteria specified ZA and AA only. Sammut et al.’s [13] study was removed as it employed a case series study design, which did not meet the eligibility criteria requiring higher levels of evidence. Martins et al. [14] was excluded because it was a systematic review and did not provide original patient data. Lastly, the study by Manzano-Moreno [15] was excluded as it was an in vitro study, whereas the criteria required research conducted on human subjects.

**Figure 1:**
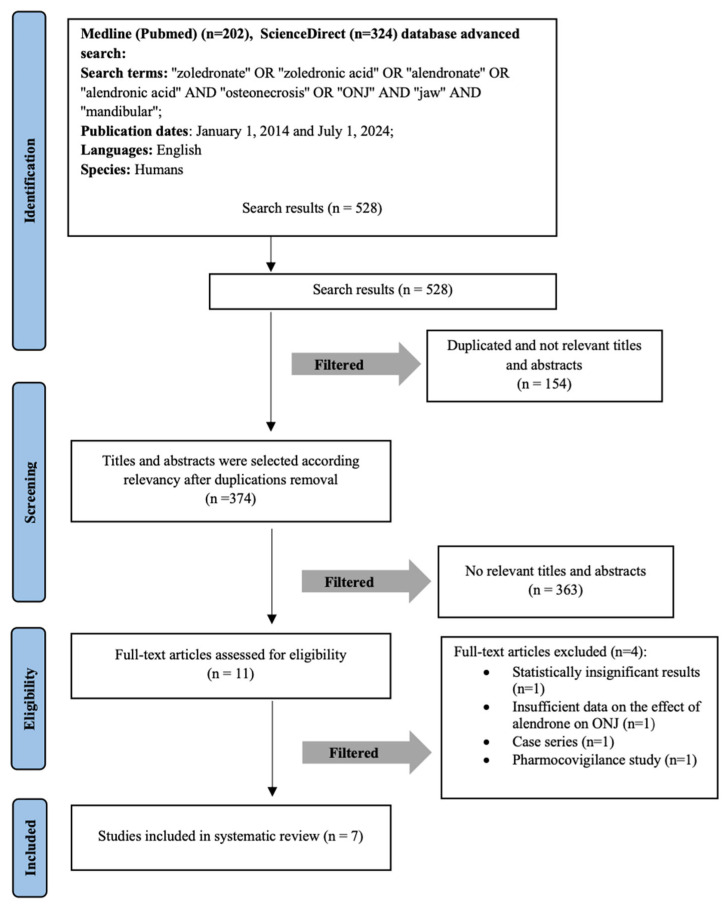
Flow diagram of studies selection according to PRISMA guidelines. Study selection according to PRISMA guidelines.

Seven full-text retrospective cohort publications were included in the systematic literature review for the deeper analysis. The detailed selection process of the scientific publications is illustrated in the methodological PRISMA diagram (Figure 1). The agreement between the two authors (A.J. and E.G.) in selecting abstracts was assessed, with a κ value of 0.91.

### 3.2. Quality Assessment of the Included Studies

The quality of the included studies is outlined in Table 3. All studies were rated as high-quality according to the JBI score: four studies [16,17,18,19] were characterized > 8, two studies [20,21] had a score of >9, and one study had a score 7/10 [22].

### 3.3. Study Characteristics

A systematic literature review included seven retrospective cohort studies with a total of 98,717 patients, of whom 78,898 were female, indicating a predominantly female patient population in six studies [16,17,18,19,20,22]. Additionally, two studies [16,20] reported that the vast majority of ONJ cases occurred in female patients. The average patient age across all studies was over 60 years, and a total of 1388 ONJ cases were identified [16,17,18,19,20,21,22].

The included studies examined the use of ZA or AA in osteoporotic patients. Specifically, four studies [16,19,20,22] focused on patients treated with AA, two studies [18,21] included patients receiving either ZA or AA, and one study [17] exclusively investigated patients treated with ZA.

A comprehensive summary of the study characteristics, including BP type, incidence, risk factors for ONJ development, and treatment duration, is presented in Table 4 and Table 5.

### 3.4. Zoledronic Acid

#### 3.4.1. The Incidence of ONJ Development

The incidence of ONJ among patients receiving ZA was analyzed in multiple studies. Chen et al. [17] found that ZA use exceeding 18 months was significantly associated with an increased risk of ONJ recurrence (*p* = 0.016), emphasizing the importance of monitoring long-term ZA therapy. Fung et al. [18] documented a median time to ONJ onset (TTO) of 2.2 years for ZA users, with a total of 218 cases recorded in their cohort study. Additionally, Amigues et al. [21] reported an incidence of 9.6 cases per 100,000 patient-years. The study also highlighted differences in ONJ occurrence based on patient populations, showing that ZA use in oncology patients was associated with a significantly higher ONJ incidence compared to those treated for rheumatologic conditions (*p* < 0.001).

#### 3.4.2. Risk and Impact of Treatment Duration on ONJ Development

Several studies examined the relationship between ZA treatment duration and ONJ risk. Chen et al. [17] further indicated that ZA use beyond 18 months significantly increased ONJ recurrence risk (*p* = 0.016), reinforcing the importance of treatment monitoring. Fung et al. [18] reported a median time to onset of 2.2 years of ZA users, highlighting a relatively rapid onset compared to other BP. Additionally, Amigues et al. linked ZA therapy to a shorter time to ONJ onset, with a median time to onset of 27 ± 22 months in oncology patients and 49 ± 22 months in rheumatology patients (*p* = 0.003).

It has also been found that the risk of ONJ in ZA-treated patients was closely linked to treatment duration and specific patient characteristics. Amigues et al. [21] demonstrated that ZA use was associated with a significantly increased ONJ risk in oncology patients, where the likelihood of ONJ development was 135 times higher than in rheumatology patients (*p* < 0.001).

### 3.5. Alendronic Acid

#### 3.5.1. The Incidence of ONJ Development

Studies investigating ONJ incidence in patients treated with AA also provided key insights. Eiken et al. [16] revealed a fourfold increase in ONJ risk among recent AA users compared to past users (*p* = 0.02), suggesting that ONJ incidence may be time-dependent. Another study by Chiu et al. [20] found a cumulative ONJ incidence of 0.55% over 12 years, corresponding to 283 cases per 100,000 patient-years. Chiu et al. [19] further noted that patients treated with AA for more than three years experienced a higher incidence rate (0.92%) compared to those treated for less than three years (0.24%, *p* = 0.002). However, Lin et al. [22], did not find a significant increase in ONJ risk among patients receiving AA within the first four years of treatment.

#### 3.5.2. Risk and Impact of Treatment Duration on ONJ Development

For patients treated with AA, ONJ risk could be influenced by both treatment duration and cumulative dosage. Eiken et al. [16] noted that ONJ risk increased significantly after more than five years of AA therapy, with long-term users exhibiting the highest risk. Chiu et al. [19] identified a 3.15 to 7.42 fold increased ONJ risk among AA users, independent of treatment duration, demographics, or comorbidities, and observed a progressive increase in ONJ incidence over time, with rates rising from 0.23% after two years of treatment to 0.92% after ten years. In contrast, Lin et al. [22] did not find a clear correlation between cumulative AA dosage and ONJ development, indicating that other factors might contribute to the onset of ONJ. However, they reported no significant increase in ONJ risk during the first four years of AA treatment, suggesting that the effects of treatment duration on ONJ risk may become more evident later in therapy.

## 4. Discussion

The systematic literature review is based on the analysis of seven publications. Although the researchers compared different types of BP—ZA (intravenous) and AA (oral)—a relationship was found in six out of the seven publications between the incidence, risk, and impact of treatment duration on ONJ development. However, other authors conducting studies have obtained different results, indicating that the risk frequency of ONJ development is influenced by other factors as well.

Several studies have identified tooth extraction (TE) as a major risk factor for ONJ in BP users. Chiu et al. [19] reported that TE increased ONJ incidence from 0.34% to 2.16% (*p* < 0.001), demonstrating a 9.6-fold higher ONJ risk regardless of BP duration. Similarly, Chen et al. [17] found that 61.3% of ONJ cases in ZA-treated patients were linked to TE. Beyond TE, other oral health factors such as periodontal or periapical disease, peri-implant disease, and denture-related trauma were associated with ONJ development in BP users. These findings emphasize the broader impact of oral health status on ONJ risk and underscore the importance of pre-treatment dental evaluations and close monitoring of patients undergoing invasive dental procedures.

Certain underlying medical conditions have been linked to an elevated ONJ risk in BP-treated patients. Eiken et al. [16] reported a higher prevalence of ONJ among AA users with rheumatoid diseases and those on proton pump inhibitors, suggesting that systemic factors may influence ONJ susceptibility. Lin et al. [22] also identified gingival and periodontal diseases as significant contributors to ONJ in patients receiving AA therapy. In contrast, Saag et al. [23] observed no ONJ cases in a cohort of 2014 AA-treated patients, who received calcium and vitamin D supplementation, indicating a potential protective role of nutritional factors. Furthermore, Amigues et al. [21] demonstrated that antiangiogenic agents used in oncology patients accelerated ONJ onset in ZA users, reinforcing the necessity of individualized risk assessment considering comorbidities and concurrent medications.

Long-term BP therapy and advanced age have been recognized as key risk factors for ONJ. Chiu et al. [20] reported that patients aged 65–80 years had a 4.14-fold increased ONJ risk, which further escalated to 5.65-fold for those over 80 years. Additionally, BP use beyond three years significantly elevated ONJ risk (OR 5.73, 95% Cl 2.967–11.044). The comparison of hazard ratios further demonstrated a strong correlation between age and ONJ risk, emphasizing the need for careful monitoring in long-term BP therapy.

The route of BP administration significantly influences the risk of developing ONJ. Intravenous BP, such as ZA, are often associated with a higher incidence of ONJ compared to oral BP like AA. This disparity is attributed to differences in pharmacokinetics, bioavailability, and patient population. Oral BP have low bioavailability; for instance, ibandronic acid exhibits a bioavailability of approximately 0.6%. After absorption, about 50% of the drug is excreted unchanged by the kidneys, while the remainder binds to the bone tissue, where it can remain for an extended period, with a half-life exceeding ten years [24]. In contrast, BP bypass the gastrointestinal tract, leading to more predictable absorption and higher bioavailability, which may contribute to the increased risk of adverse effects such as ONJ.

Martins et al. [14] reported an overall ONJ incidence of 2.7% (95% Cl: 0.9–5.2%), with a significantly higher risk among intravenous BP users (6.9%) compared to oral BP users (0.2%). Pelaz et al. [24] similarly found that ONJ was more common in ZA-treated patients, even at lower cumulative doses. Amigues et al. [21] further confirmed that ONJ incidence with ZA was nearly double that of AA (9.6 vs. 5.1 per 100,000 patient-years, *p* < 0.001).

Intravenous BP are frequently prescribed in higher doses for cancer patients in order to manage bone metastases and hypercalcemia—conditions that inherently carry a higher risk for ONJ [24]. Conversely, oral BP are commonly used in lower doses for osteoporosis treatment in non-cancer patients, who generally have a lower baseline risk for ONJ [21]. Additionally, Martins et al. [14] observed that even at lower cumulative doses, ZA-treated patients exhibited a higher ONJ incidence than those on AA, reinforcing the significance of BP potency and cumulative exposure.

Key ONJ risk factors include dental procedures, corticosteroid use, and pre-existing dental conditions [21]. Preventive strategies involve pre-treatment dental assessments, maintaining excellent oral hygiene, and considering a temporary discontinuation of the medication for high-risk patients [24].

These findings suggest that intravenous BP, particularly ZA, carries a significantly higher and earlier ONJ risk compared to oral BP like AA. Additionally, this review found that ONJ associated with AA can develop as early as one year of use, while ONJ related to ZA may appear as early as five months of use, underscoring the importance of long-term monitoring and individualized risk assessment. As ONJ is a multifactorial condition, individualized patient risk assessment and early intervention strategies are key factors to optimizing clinical outcomes.

### Limitations

This systematic literature review has several limitations that may impact the accuracy of its findings. The variability in study designs, including differences in study populations, methodologies, and ONJ definitions, introduces heterogeneity that could influence the comparability of results. Additionally, differences in BP use duration and the retrospective nature of some studies may contribute to selection and reporting biases, influencing ONJ incidence accuracy.

Another limitation is ONJ underreporting, which may lead to an underestimation of true incidence. Many studies relied on hospital records and diagnostic codes, potentially missing undiagnosed or outpatient cases. Additionally, confounding factors, such as underlying health conditions, dental case variations, and patient adherence, were not always fully addressed. In addition, the diversity in BP types, doses, and administration routes further complicates drawing definitive conclusions. Intravenous ZA was more frequently linked to ONJ than oral BP, but differences in cumulative exposure and concurrent treatments were not consistently analyzed.

Future research should focus on standardizing methodologies, expanding study populations, and exploring BP interactions with other risk factors, particularly genetic predisposition, medication combinations, and preventive dental care strategies.

## 5. Conclusions

This systematic review highlights that ONJ associated with AA can develop as early as 1 year, while ZA may induce ONJ within 5 months of use, with ZA posing a higher overall risk and earlier onset compared to AA. The findings reinforce that ZA carries a greater ONJ risk emphasizing the need for careful patient monitoring and preventive dental measures.

## Figures and Tables

**Table 1:** Focus question development according to the PICO model.

Component	Description
**Population (P)**	Patients with OP taking BPs medications such as ZA or AA.
**Intervention/ Exposure to a risk factor (I)**	The effect of ZA or AA on the occurrence of ONJ.
**Control (C)**	Patients without OP and patients who do not use BPs.
**Outcome (O)**	The occurrence of ONJ in patients receiving BP therapy.

**AA**—alendronic acid; **BP**—bisphosphonates; **ONJ**—osteonecrosis of the jaw; **OP**—osteoporosis; **ZA**—zoledronic acid.

**Table 2:** The JBI Critical Appraisal Checklist for cohort studies.

Question Number	Defined Question
**Q1**	Were the two groups similar and recruited from the same population?
**Q2**	Were the exposures measured similarly to assign people to both exposed and unexposed groups?
**Q3**	Was the exposure measured in a valid and reliable way?
**Q4**	Were confounding factors stated?
**Q5**	Were strategies to deal with confounding factors stated?
**Q6**	Were the groups/participants free of the outcome at the start of the study (or at the moment of exposure)?
**Q7**	Were the outcomes measured in a valid and reliable way?
**Q8**	Was the follow-up time reported and sufficient to be long enough for outcomes to occur?
**Q9**	Was follow-up complete, and if not, were the reasons to lose to follow-up described and explored?
**Q10**	Were strategies to address incomplete follow-up utilized?
**Q11**	Was appropriate statistical analysis used?

**Table 3:** Results from cohort studies based on JBI Critical Appraisal Checklist.

Study	Study Design	Checklist										
		Q1	Q2	Q3	Q4	Q5	Q6	Q7	Q8	Q9	Q10	Q11
Eiken et al., 2017 [16]	Cohort study	+	+	+	+	+	+	+	+	?	?	+
Chen et al., 2021 [17]	Cohort study	?	+	+	+	+	+	+	+	+	-	+
Fung et al., 2016 [18]	Cohort study	+	+	?	NA	+	+	+	?	+	+	+
Chiu et al., 2014 [20]	Cohort study	+	+	?	+	+	+	?	+	+	+	+
Chiu et al., 2018 [19]	Cohort study	+	+	?	+	+	+	+	+	+	?	+
Lin et al., 2014 [22]	Cohort study	+	+	?	+	-	+	?	+	+	+	+
Amigues et al., 2023 [21]	Cohort study	+	+	+	+	+	+	+	+	?	+	+

? = unclear; + = yes; - = no; NA = not applicable.

**Table 4:** The incidence of ONJ.

Type of BP	Author (Year)	Follow-Up (Years)	Number of Patients	Number of Females	Mean Age	Number of ONJ Cases	Females with ONJ
**AA**	Eiken et al., 2017 [16]	6.8	61.990	51.558	74.9	**107**	85
	Fung et al., 2016 [18]	8	349	247	≥60	88	-
	Chiu et al., 2014 [20]	12	7.332	6.485	74.9	40	39
	Chiu et al., 2018 [19]	12	7.625	6.356	73.7	26	-
	Lin et al., 2014 [22]	6	18.030	14.213	78.1	25	-
	Amigues et al., 2023 [21]	10	1.039	-	70	**188**	-
**ZA**	Chen et al., 2021 [17]	5	58	39	63.2	58	-
	Fung et al., 2016 [18]	8	349	247	≥60	**218**	-
**ZA^1^**	Amigues et al., 2023 [21]	10	2.294	-	68	70	-
**ZA^2^**	Amigues et al., 2023 [21]	10	1.103	-	66	**568**	-

**AA**—alendronic acid; **BP**—bisphosphonates; **ONJ**—osteonecrosis of the jaw; **ZA**—zoledronic acid; **ZA^1^**—zoledronic acid for rheumatology patients; **ZA^2^**—zoledronic acid for oncology patients.

**Table 5:** Risk factors and time duration for ONJ development in AA or ZA users.

Type of BP	Author (Year)	Risk Increase with BP Duration	ONJ Dependence on BP Timing
**AA**	Eiken et al. [16]	>5 years	Positive
	Fung et al. [18]	6 years	Positive
	Chiu et al. [20]	>3 years	Positive
	Chiu et al. [19]	>1 year	Positive
	Lin et al. [22]	-	Negative
	Amigues et al. [21]	69 ± 54 months	-
**ZA**	Chen et al. [17]	>18 months	Positive
	Fung et al. [18]	2.2 years	Positive
**ZA^1^**	Amigues et al. [21]	49 ± 22 months	Positive
**ZA^2^**	Amigues et al. [21]	27 ± 22 months	Positive

**AA**—alendronic acid; **BP**—bisphosphonates; **ONJ**—osteonecrosis of the jaw; **ZA**—zoledronic acid; **ZA^1^**—zoledronic acid for rheumatology patients; **ZA^2^**—zoledronic acid for oncology patients.

## Data Availability

Data described in the manuscript, code book, and analytic code will be made available upon request pending application and approval from the corresponding author.

## Abbreviations

OP	Osteoporosis
ONJ	Osteonecrosis of the jaw
BP	Bisphosphonate
ZA	Zoledronic Acid
AA	Alendronic Acid
MRONJ	Medication-related osteonecrosis of the jaw
PRISMA	Preferred Reporting Item for Systematic Review and Meta-Analyses
JBI	The Joanna Briggs Institute
TTO	Median time to ONJ onset
TE	Tooth extraction
